# Framing ethical issues associated with the UK COVID-19 contact tracing app: exceptionalising and narrowing the public ethics debate

**DOI:** 10.1007/s10676-022-09628-z

**Published:** 2022-01-24

**Authors:** G. Samuel, F. Lucivero

**Affiliations:** 1 Department of Global Health and Social Medicine, King’s College London, Bush House, Strand, London, UK; 2 Ethox Centre and Wellcome Centre for Ethics and Humanities, Oxford University, Oxford, UK

**Keywords:** Ethics, Advocacy, Contact tracing, Bioethics, Digital, Privacy

## Abstract

This paper explores ethical debates associated with the UK COVID-19 contact tracing app that occurred in the public news media and broader public policy, and in doing so, takes ethics debate as an object for sociological study. The research question was: how did UK national newspaper news articles and grey literature frame the ethical issues about the app, and how did stakeholders associated with the development and/or governance of the app reflect on this? We examined the predominance of different ethical issues in news articles and grey literature, and triangulated this using stakeholder interview data. Findings illustrate how news articles exceptionalised ethical debate around the app compared to the way they portrayed ethical issues relating to ‘manual’ contact tracing. They also narrowed the debate around specific privacy concerns. This was reflected in the grey literature, and interviewees perceived this to have emerged from a ‘privacy lobby’. We discuss the findings, and argue that this limited public ethics narrative masked broader ethical issues.

## Introduction

In response to the COVID-19 pandemic, in 2020 policymakers across the world announced that they would be developing contact tracing applications (apps) to assist with traditional (‘manual’) modes of tracing individuals who may have been exposed to the virus, by automating the process of both exposure measurement and contact notification. This automation was perceived to speed up the process, especially given the virus was infectious during the period leading up to and immediately after the start of symptoms. This would also alleviate the time-consuming nature of contact tracing, allowing an interruption in the chain of transmission for both symptomatic and asymptomatic contacts of an index case, by instructing them to self-isolate even though they may not have symptoms. In some countries, such as China, Taiwan and South Korea, apps were designed to use GPS to track the location and other metadata, such as credit card transactions from their citizens, to trace movements and potential exposures to the virus (Huang et al., 2020; Steinbrook, 2020). In other countries, such as the UK and many European countries, apps were developed to rely on Bluetooth-based technology. This was deemed more privacy-preserving compared to the use of GPS-based technology because it infers the distance between two phones rather than estimating their geographical location. Bluetooth-based technologies allow the collection of random codes that are shared by devices in close proximity for a specific length of time. When a positive case is registered on a device’s app, all devices which have recently shared a code will receive an alert to self-isolate.

In spring 2020, at the time many countries were developing their contact tracing apps, considerable debate was generated over the ethical issues perceived to arise from the use of such a technology. In particular, debates centred around the choice of app design (Anderson, 2020; Kahn & Johns Hopkins Project on Ethics and Governance of Digital Contact Tracing Technologies, 2020; Lucivero et al., 2020; Sharon, 2020), as well as the governance structure established to oversee the technology’s development, and the openness and transparency with which this governance process was communicated to the public (AdaLovelace Institute, 2020b; Kahn & Johns Hopkins Project, 2020; Kerr et al., 2020; Lucivero et al., 2020; Parker et al., 2020).

This paper’s aim is to contribute to work that takes this ethics debate as an object for sociological study (Hedgecoe, 2010). Such work—sometimes called the sociology *of* ethics (Cribb, 2020; Haimes, 2002; Pillay, 2014)—charts the social forces that help shape ethical issues, examining the ways in which certain ethical debates are privileged, whilst others tend to be marginalised (Williams & Wainwright, 2013). Ethics debate, then, is viewed as a sociological/social phenomenon, such that there may be a range of ethical issues associated with any particular topic, but that it is social processes rather than a priori ethical value that will determine which particular issues become dominant in scholarly, policy and/or public discussions. Key to this literature is an acknowledgement of the importance in understanding how social processes work to define what counts as ethical. In doing so, for any particular topic of exploration, this literature can expose missing debates or uncover problematic implicit assumptions, and indeed this is the main value of this approach. Work in this area has explored how ethics is framed in global health ethics policy documents (Brisbois & Plamondon, 2018), the scientific literature (Hedgecoe, 2010), research ethics (Emmerich, 2020), ethics guidelines (Jobin et al., 2019) and the professionalisation of ethics (Wilson, 2014). Furthermore, when applied to the public arena, the sociology of ethics literature also intersects literature that focuses on ‘performative ethics’. This term has been appropriated by scholars and disciplines in various ways, but ultimately relates to the idea that narratives (including those in the public arena) have an important role to play in creating and shaping moral futures through social processes (Edwards et al., 2015; Hoover, 2019; Miller, 2019; Santis & Zavattaro, 2019).

This article explores the ethics discussion associated with the UK COVID-19 contact tracing app that occurred in the ‘public ethics narrative’. Evans (2006) has argued that this narrative is composed primarily of the news media and broader public policy discussions, both of which have an important role to play in public (ethics) debate. This is because research has repeatedly documented the agenda-setting role of the news media in influencing public debate (Dearing & Rogers, 1996; Jann & Wegrich, 2006). While many forms of media (news, social media, other etc.) have a role in informing public audiences and debate, the news media is a prominent producer of public narratives and narrator to public audiences (Henderson & Hilton, 2018; Miller et al., 1998), and furthermore, was a key platform of public information during the pandemic (De Coninck et al., 2020). As such, an analysis of news media and broader public policy, with a specific focus on ethics, will help us to understand the construction of the public ethics narrative.

This article’s research question was, how did UK national newspaper news articles and UK grey literature frame the ethical issues associated with the UK COVID-19 app between its inception until June 2020, and how did stakeholders associated with the development and/or governance of the app reflect on this? We examined the predominance of how different ethical issues were discussed in news articles and grey literature, and triangulated this using our interview data. Our findings illustrated how the news articles we analysed, exceptionalised and narrowed the ethical debate around the app compared to the way they portrayed the ethical issues relating to manual contact tracing.

## The UK Covid-19 contact tracing app

In early spring 2020, a UK contact tracing app was developed by NHSX, the unit of the NHS (National Health Service) responsible for digital innovation. Alongside concerns raised in other countries about analogous apps, a wide range of ethical issues were voiced in the scholarly literature and public domain. These issues included: issues of privacy, surveillance, data protection, and the increased involvement of Big Tech corporations in public health solutions (Lucivero et al., 2020); questions around the nature of public–private collaborations in terms of who would have access to any data collected by the app, and under what conditions; appropriate oversight, including institutional responsibility and trustworthiness, and openness and transparency (AdaLovelace Institute, 2020a, 2020b; Lucivero et al., 2020; NHSX app Ethics Advisory Board, 2020; Parker et al., 2020); the uptake and feasibility of a voluntary contact-tracing app and the necessity for clear, transparent language in consent agreements (Bengio et al., 2020); the potential value of the app (the need for appropriate evaluation protocols to measure actual health value in practice) (NHSX app Ethics Advisory Board, 2020); and concerns that contact tracing apps could reinforce digital divides, exacerbate health inequalities, or unfairly discriminate against particular groups (Gasser et al., 2020; Morley et al., 2020; NHSX app Ethics Advisory Board, 2020; Wright, 2020).

However, in the UK, by far the most prominent criticism was related to the decision to develop a contact tracing app using a centralised model of data collection. In this model, the data about the number of devices a user had been in contact with, plus the signal strength and signal duration, would be transmitted back to a central, anonymised database when a user alerted an app that they may have had COVID-19 symptoms. UK public health experts hoped that analysis of this data would provide insight into how the virus was spreading, as well as allow intervention to contain the virus (e.g. by altering the performance of the app in different parts of the country depending on how the virus was being transmitted). However, many commentators deemed this model ‘privacy-diminishing’ in comparison to what they perceived to be the more ‘privacy enhancing’ decentralised model adopted in many European countries (Abeler et al., 2020), which was based on an Application Programming Interface (API) provided by Google and Apple. In this latter model, when a user alerts an app that they have tested positive to COVID-19, the random codes shared by devices predominantly remain on people’s phones. Privacy advocates in the UK were worried about access to data in the centralised model, as well as the data being re-purposed for other uses (Anderson, 2020).

Nevertheless, the UK NHSX app was trialled on the Isle of Wight in May/June 2020. However, the trial was halted in June 2020, reportedly due to technological issues. The app was later remodelled by NHS Test and Trace using an API provided by Google and Apple, and launched in England and Wales in September 2020. At the time of writing, the app has been downloaded more than 20 million times. Subsequent research suggested the trial was associated with a marked reduction in virus spread (Kendall et al., 2020).

## Methods

### News article data collection

**Fig. 1:**
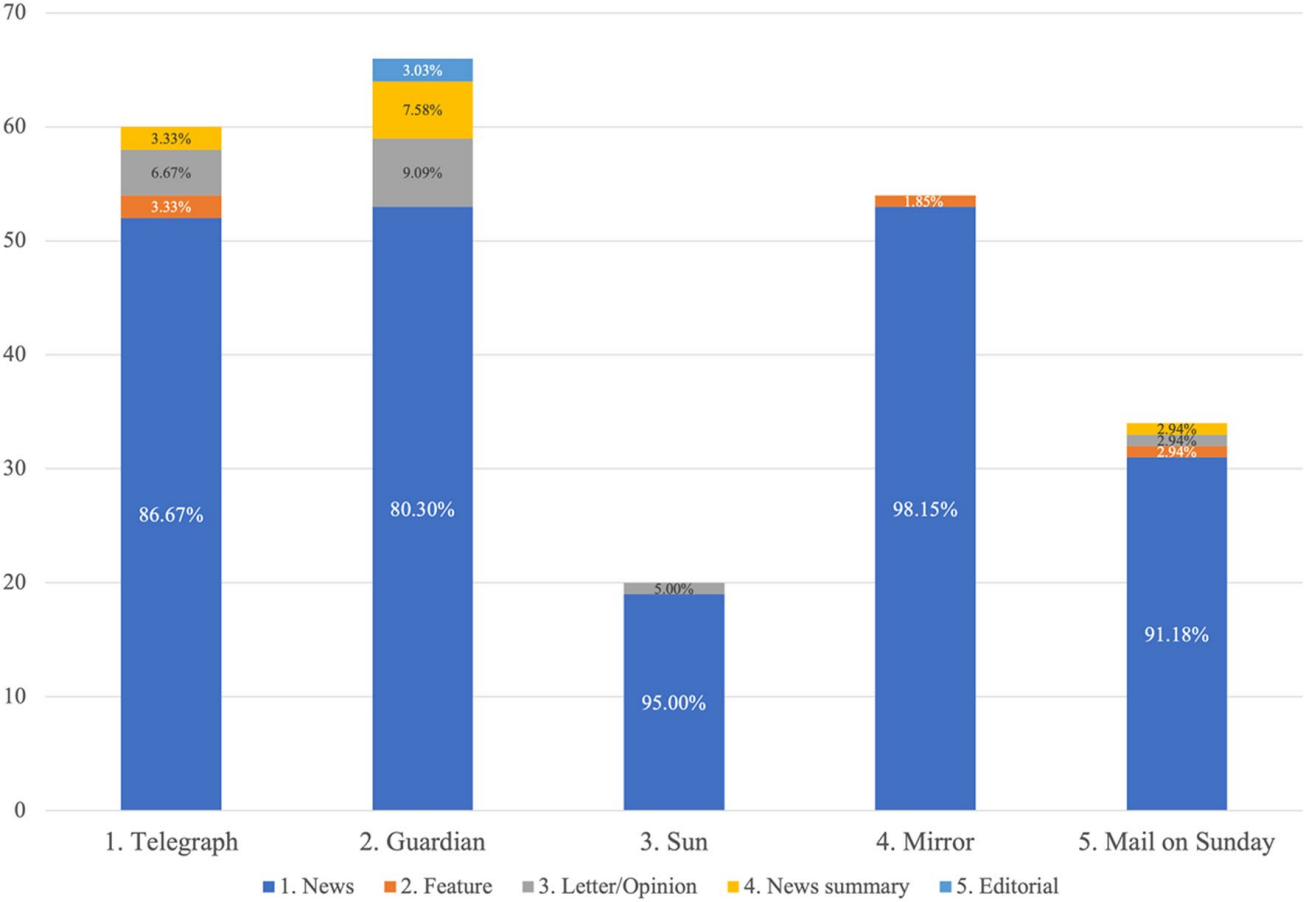
Number of articles per newspaper which discussed the app. Also includes percentage of article type

Headlines and lead paragraphs of UK national newspapers were searched in Nexis on 26th June 2020 using the search terms (‘digital tracking’ or ‘digital tracing’ or ‘app’) and (corona* or COVID*) and NHS* (all dates). Nexis is a comprehensive, online news database commonly used in social scientific research, which contains newspapers and other news articles from around the world. The sample collection date represented the timeframe soon after the app trial was halted. It also included the launch of the UK government’s test and trace programme. The analysis was focused on five specific newspapers: the telegraph.co.uk, The Guardian, the MailOnline, the mirror.co.uk and thesun.co.uk. These represented UK national newspapers which have an offline and online presence, and represent different political leanings (left/right) and readership (low, mid, high socio-economic status).^1^ 1230 articles were retrieved. Articles were removed if duplicate, or the headline was not relevant (including not focusing on contact tracing). Articles were retained if they included a substantial discussion (at least a paragraph) on either the app or the NHS test and trace programme. 259 qualified for further analysis, and 234 focused specifically on the app (Table 1). News article demographics are illustrated in Fig. 1.

**Table 1:** Number and percentage of articles mentioning the app, test & trace, and overlap between the two

Type	Number of articles	Percentage
Articles mentioning the app	234	90.35
Articles mentioning test and trace	122	47.10
Overlap (both mentioned)	97	37.45
Total number of articles	259	100

### Grey literature data collection

An analysis of grey literature was used as a proxy for the public policy debate, as it included various statements, reports and open letters written by the UK government and other commentators (Table 2). A Google search at the end of June 2020 included the keyword strings (a) “(corona* OR covid* OR SARS-COV-2) AND NHS* AND ("digital tracking" OR "digital tracing" OR "tracing app" OR “contact tracing”). Results were sorted by relevance. All retrieved webpages were checked for pertinent articles until links were no longer relevant. Articles were included if they discussed the UK NHSX app and if ethics was a major or significant component of the discussion. 103 weblinks were reviewed. Snowballing and additional website searches (e.g. on Hansard) identified a further 255 weblinks that were reviewed. Further, four articles were added to the sample that were not picked up in searches. Exclusion criteria were then applied, including if (1) they were academic papers or blog posts; (2) they did not focus specifically or predominantly on the NHSX app; or (3) they discussed contact tracing in the UK but did not focus on the NHSX app. 48 articles were deemed as fitting the inclusion criteria. A range of different actors authored these articles, including the government, NHSX, government advisors, and independent commentators (Table 2).

**Table 2:** Source of grey literature articles used in analysis

Source	Description	Number of articles
NHSX	NHSX statements about the announcement or functioning of the app	6
Government	UK government official statements about the app, including letters addressing questions to the Secretary of State for Health and Social Care and House of Commons	8
Parliament committees	Evidence from committee meetings, reports and letters written by the Joint Committee on Human Rights and the Science and Technology Committee	8
ICO & NCSC^a^	Statements and reports published by the organisations, and letters to address specific questions	7
Independent commentators^b^	Statements, reports, open letters, and written evidence to parliament made by various professional organisations, legal and security experts	17
Government advisors	Reports and letters from the Ethics Advisory Board and SAGE, advisors on app and general tracing programme	2
Total		48

^a^*ICO* Information Commissioner’s Office, *NCSC* National Cyber Security Centre; involved with the app’s development

^b^Independent commentators included academics, predominantly those with expertise in privacy and security, and NGOs and other organisations (e.g. Ada Lovelace Institute, Liberty, Open Rights Group)

#### Coding

A deductive approach was used for coding. A coding framework was first developed inductively and deductively. Codes were deductively drawn from key issues emerging in the literature and policy debates associated COVID-19 apps, and from the ethics literature on emerging technologies more generally. Codes also included demographic codes, including date and type of publication, and newspaper/author and/or institutional source. News articles and grey literature were then scanned independently by two researchers (GS and a research assistant; a different research assistant for the grey literature and newspaper analysis), to inductively identify additional codes for each analysis. For each analysis, these inductive codes were combined with the deductive codes. Each framework was then applied to a sample of 10 news articles, and 5 grey literature articles, respectively, by two independent researchers (GS and a research assistant; a different research assistant for the grey literature and newspaper analysis). For each analysis (grey literature and news articles), coding was compared between researchers; additional codes were added where necessary, and others were modified or removed (Amann et al., 2021). At this stage, researchers discussed in-depth, inconsistencies in coding that had emerged. The two researchers then independently applied the up-dated coding frameworks to a different sample of newspaper and grey literature articles, as before. Codes were again compared in-depth, to ensure coding was consistent. The final coding framework for both the grey literature and news article analysis is described in Table 3. Ethical issues were defined broadly, but excluded social/behavioural issues (for example, misuse, and hacking/phishing scams; will social infrastructures be able to accommodate the likely rise in testing; will people use/respond to the app; the number of people needing to download the app for it to be effective) and technical issues (for example, the technology not working or needing to be kept up to date, the app not being compatible across countries, or not taking into account enough symptoms, Bluetooth issues, phone battery usage etc.). Discrepancies in coding themes between the grey literature and the news media are highlighted, and were due to refinements in the deductive coding structure during the early stages of inductively reading each of the documents (grey literature vs news articles). As such, differences reflect the information contained within each of our samples.

**Table 3:** Grey literature and news article coding for ethical issues associated with the UK NHSX COVID-19 contact tracing app

Code	Description of code, where relevant/needed	Coded in grey literature?	Coded in news articles?
Data and privacy concerns including centralised/de-centralised system	Centralised/de-centralised system; personal data; anonymisation versus pseudonymisation; re-/identification of individuals; data access; data usage and processing; data retention; right to access, right to be forgotten; concerns about hacking; security	Y	Y^a^
Public–Private corporations	e.g. in terms of app development or testing (including Apple/google etc.)	Y	Y
Liberty/freedom/anonymity	Human rights; infringing our liberties; right to privacy and family life; nobody penalised for forgetting phone, not charging, not downloading	Y	Y
Discrimination and equity of access	Vulnerable, elderly, children—digitally excluded; no access to employment and income; employment decisions based on app information; vulnerable, people with disabilities; right to non-discrimination; discrimination due to profiling	Y	Y^b^
Consent	Including voluntariness	Y	Y
Openness/transparency	Including from the government to ethics boards or from the government to local government, or from the government to the public	Y	Y
Importance of public confidence/trust	Communities suspicious of app or afraid of implication. Also positive, e.g. going to work because trust in the app; also (lack of) trust in test and trace	Y	Y
Time limitation	When the app will close, time limit on data	Y	Y
Resource allocation	For app: cost of app; money be better spent elsewhere. For test and trace: workers paid to do nothing/cost to the taxpayer; cost to local councils	Y	Y
Over surveillance/biosecuritisation/‘Big Brother’	Mission creep; purpose limitation	Y	Y
Public engagement	Needing or having public engagement. Need for better communication with the public	Y	Y
Global issues	References to other countries	Y	Y
Child safeguarding	Whether appropriate for a child to download the app	Y	Y
Effectiveness within broader context of test & trace strategy	As described	Y	N
Timeliness of response	Need to act urgently	Y	N
Accountability	As described	N	Y
Independence	Biased/non-independent developers	N	Y

^a^Codes split into (A) Centralised/de-centralised systems and associated privacy concerns/for test & trace, privacy (B) Data storage and access, data security, amount of data collected

^b^Codes split into (A) discrimination & (B) equity (equal access to the app e.g. elderly excluded, primary school children don’t use phones

For each code/sub-code, articles were either coded as 0, which equaled no mention of the issue, 1 (brief mention), 2 (elaborated discussion of the issue; 2–10 sentences depending on size of article), or 3, which signified the issue was the main aspect of the article. Furthermore, in the news media analysis, distinctions were made as to whether an ethical issue was referred to in relation to the app, or to the wider test and trace scheme.

For each analysis, one researcher completed the coding, with the other researcher (GS) randomly checking the coding in a sample of every 10th newspaper article (n = 25; approximately 10%), and every 5th grey literature article (n = 9; approximately 20%). GS also read the grey literature and newspaper articles. While inter-reliability scores were not developed, coding rigour was ensured with in-depth discussions being continually held between the researcher conducting the coding and GS, to ensure any concerns or uncertainties were discussed and resolved. Furthermore, when coding, careful attention was paid to the framing of the issues, as well as how the coding categories were applied. This was particularly because categorisation is a social practice, with issues in practice being messy, overlapping and interrelated. This approach also allowed the researchers to reflect in more detail beyond the categorisation processes, as well as on the main themes and issues emerging.

### Professional stakeholder interviews

Eight interviews were conducted June–August 2020 with those involved in the app’s development or governance, those who had an app consulting role, or those who sat on the COVID-19 app Ethics Advisory Board. Recruitment was via email address identification online and snowballing. Interviews were conducted by GS (n = 5), FL (n = 1), and other collaborators (n = 2), via telephone/online, and digitally recorded. Interviews lasted 57–180 min. Interviews were part of a broader project exploring interviewees’ practices, views and experiences associated with the app’s development and governance. During interviews, interviewees shared a range of views related to the media’s and grey literature’s reporting of the app, and these reflections are reported here (see “Appendix”).

#### Analysis

Interview analysis was inductive and interactive (Strauss, 1987). Authors read the transcripts and discussed findings at two virtual meetings (along with two non-authors). GS then coded transcripts and generated themes. Themes were discussed with FL and presented in the findings. Given the political context of the app development, and the need to maintain confidentiality, no demographic information is provided about interviewees. Furthermore, where required, small changes have been made to some interviewee extracts to hide identities; care was taken to ensure changes did not detract from meanings generated.

## Findings

Findings describe the predominant themes that emerged across the news article and grey literature analysis, as well as our stakeholder interviews. These included the prominence of reporting of ethical issues associated with the contact tracing app; the prominence of debate revolving around the ethical distinctions between the centralised versus decentralised app data collection approaches as they pertained to privacy issues associated with the data collection, storage and use; and the lesser extent to which other ethical concerns related to the app technology were reported and discussed, particularly around those associated with the technology’s use.

### The ethics narrative: the pre-dominance of ‘privacy enhancing’ versus ‘privacy diminishing’

**Fig. 2:**
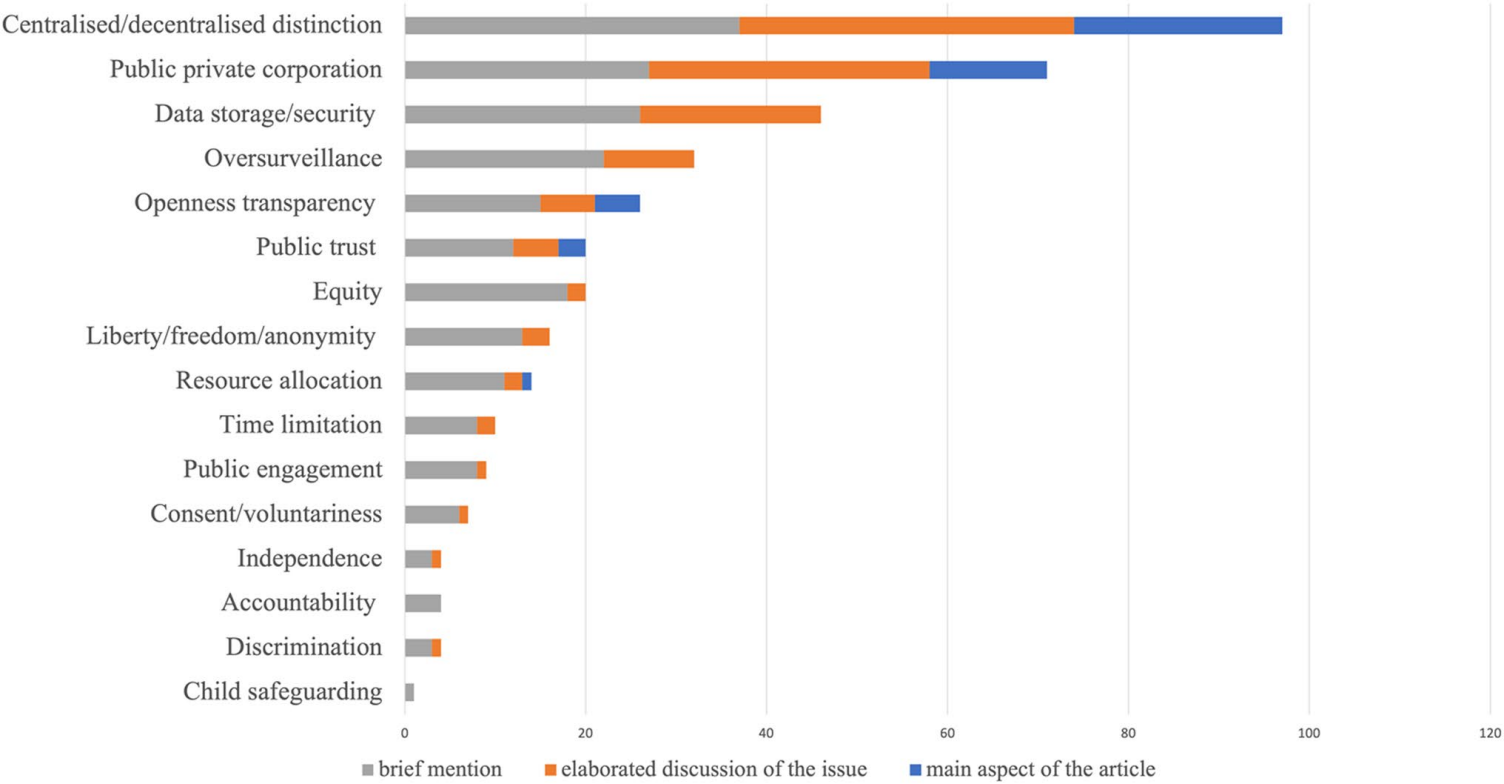
Number of mentions of each ethical issue concerning the app in the news articles, and extent to which the issue was discussed

**Fig. 3:**
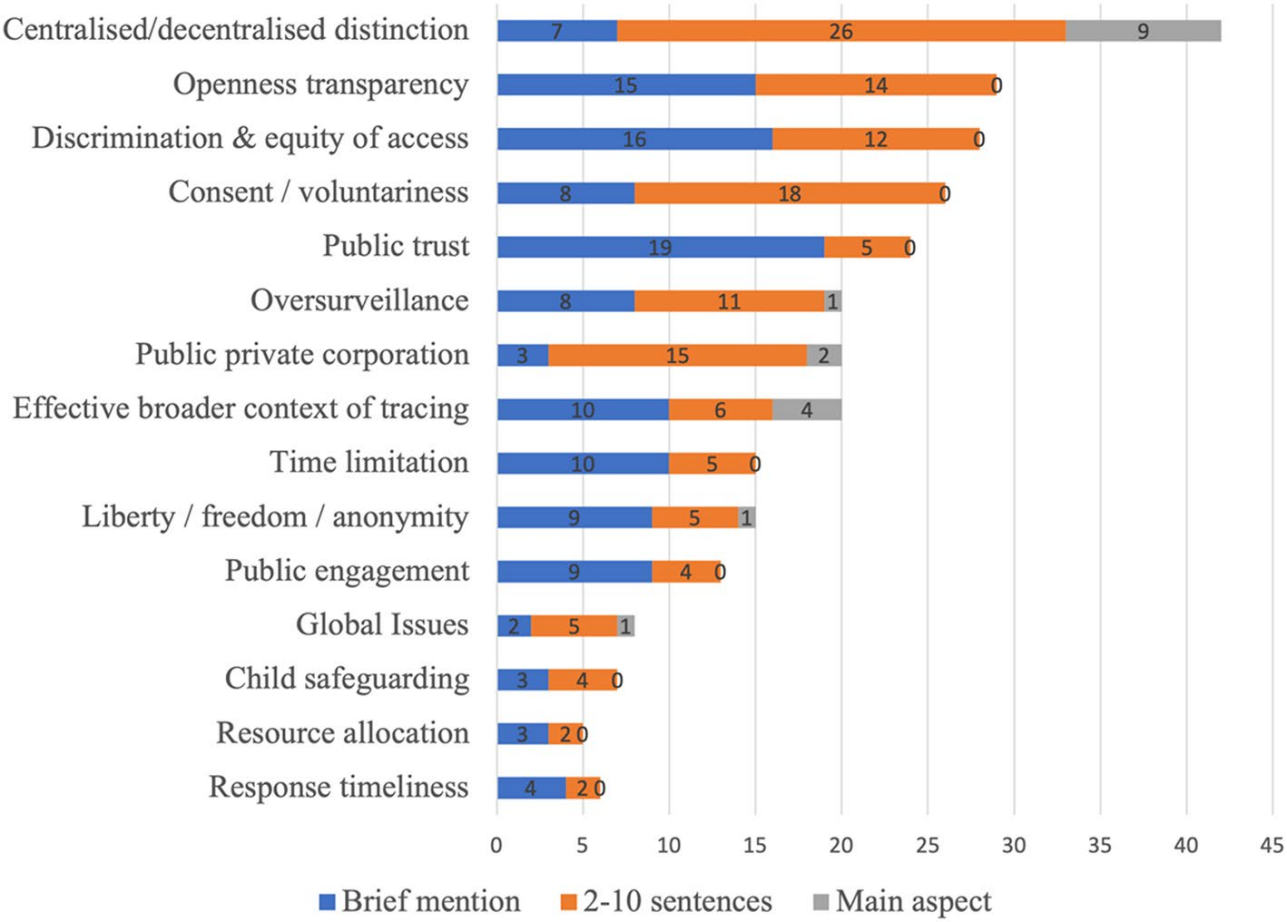
Number of mentions of each ethical issue concerning the app in the grey literature, and extent to which the issue was discussed

News articles, grey literature and stakeholder interviewees all emphasised how ethics debate about the app revolved predominantly around the ethical distinctions between the two types of app data collection models (centralised versus decentralised), as they pertained to privacy issues associated with data collection, storage and use. Figures 2 and 3 show how ethics debate about centralised versus decentralised data collections models, as well as the associated privacy issues, were the most prominent ethical issues discussed in both the news and grey literature. Debate included issues related to the security of a centralised database model in terms of potential data breaches and/or fraud; how data would be stored, processed and retained; and how data would be safeguarded against future de-anonymisation and data misuse for unspecified purposes. Over-surveillance was sometimes discussed making analogies to ‘Big Brother’, and focused on the issue of the government having access to large amounts of data on its citizens and being able to track or identify individuals. Individual rights were also discussed with relation to the compromises people would have to make regarding their liberties and rights to privacy if they used the app. Particularly in the grey literature, government and NHSX publications emphasised that a centralised approach meant better planning of regional healthcare resources, and the effective spotting of behavioural issues such as unintentional or purposeful reporting of false symptoms. However, other sources, including the Information Commissioner’s Office (ICO), and other academics and commentators, suggested a decentralised approach would be better from the perspective of data protection, and raised the question of a trade-off between the potential benefits to healthcare and the associated risk to privacy and human rights violations. Comparisons between centralised and decentralised models seemed to intensify with time, when evidence from other countries emerged and the testing of the centralised NHS app showed various defects. Here, issues focused on the government’s and NHS’s capabilities to store data securely in a centralised model, and to protect it from security breaches and function creep by both public and private partners.

The polarisation of debates around centralised and decentralised models of data collection worried nearly all stakeholder interviewees, who explained that the privacy and data protection concerns associated with the decentralised/centralised distinction represented a false dichotomy of ‘privacy enhancing’ versus ‘privacy diminishing’; decentralised models of data collection could be data insecure and vice versa:

you can build a really terribly insecure decentralised app that blows everybody’s privacy. And you can build a really secure centralised app that protects everybody’s security..[and]..the public narrative was just ridiculous. It was no basis in fact, some of the things that were said as evidence….were just factual nonsense (interviewee 8).

### Driving the narrative: concerns about ‘lobbying privacy’

**Fig. 4:**
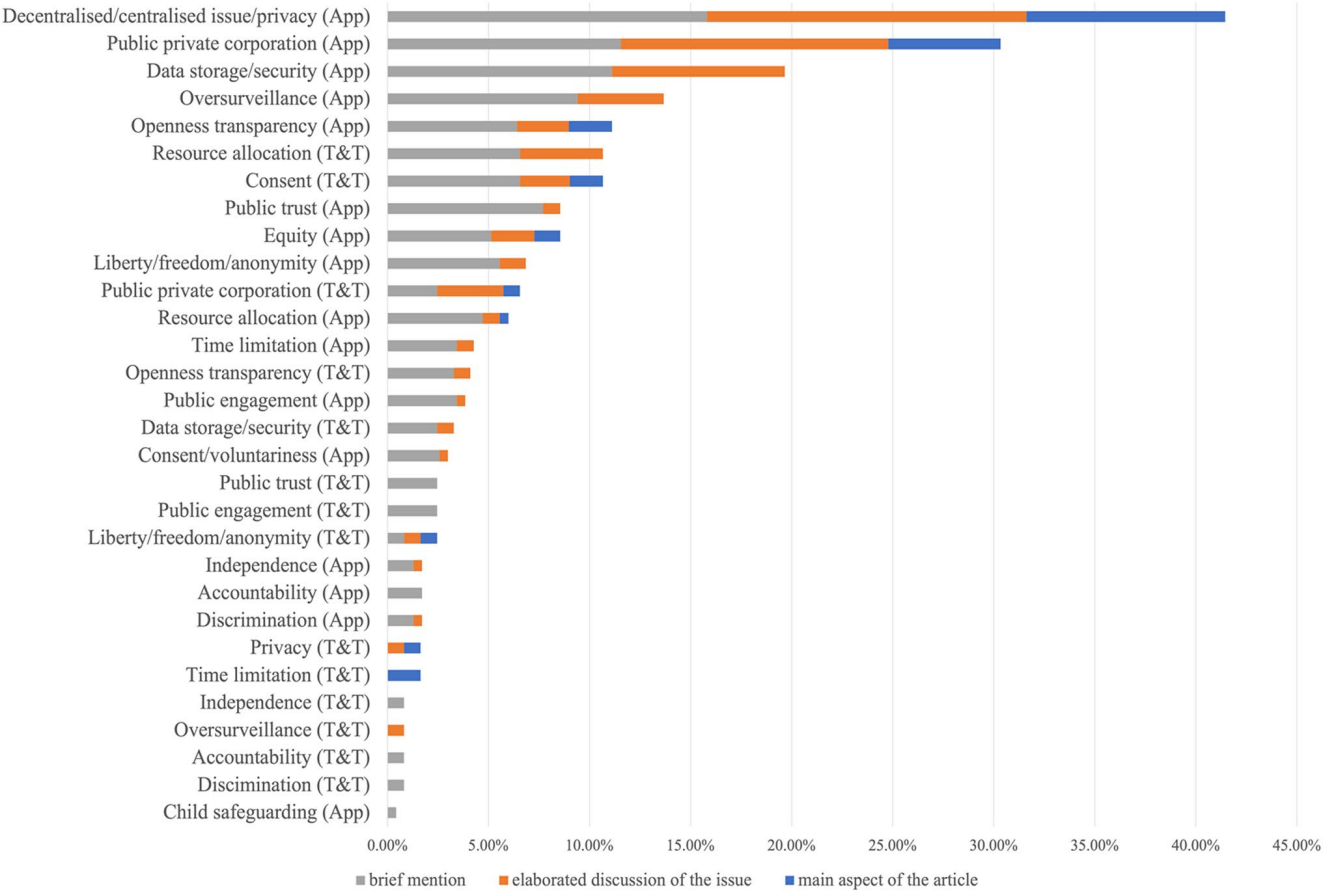
Percentage of how much each ethical issue concerning either the app, or the test and trace programme, appeared in news articles, and extent to which the issue was discussed. Each ethical issue was categorised as referring to either the app or to the wider test and trace programme. For each ethical issue referring to the app, and for each ethical issue referring to the test and trace programme, we determined the percentage that the ethical issue was mentioned as a proportion of the total number of articles mentioning the app or the test and trace programme, respectively

Our analysis suggested there was a technological exceptionalism in the framing of the app’s associated ethical issues in the news articles, with ethical issues being more discussed when reporting on the app than when articles referred to the test-and-trace programme in general. Specifically, during the coding process each ethical issue was categorised as referring to either the app or to the wider test and trace programme. For each ethical issue referring to the app, and for each ethical issue referring to the test and trace programme, we determined the percentage that each ethical issue was mentioned as a proportion of the total number of articles mentioning the app or the test and trace programme, respectively (Fig. 4). Figure 4 illustrates how, looking at these percentages, out of the top ten most prominent ethical issues mentioned, eight of the ethical issues were coded as referring to the app (signified by ‘(app)’), and only two were associated with the test and trace programme (signified by ‘(T&T)’). This technological exceptionalism seen in the newspaper articles was despite a number of stakeholder interviewees emphasising that there were just as many privacy issues in general associated with manual contact tracing compared with those related to the app. For example, interviewee 4 remarked that during contact tracing for a range of infectious diseases, it ‘literally asks…who are all your sexual partners over the last few weeks, what are their names and phone numbers, every single person you’ve been in touch with?’. Interviewee 7 echoed that while ‘the manual approach has some natural protection involved in that if someone wants to maintain privacy, they don’t have to reveal those to [a contact tracer]’. Equally, some interviewees stressed that digital tracing involved linking ‘ephemeral IDs’ rather than ‘names and numbers’ (interviewee 2).

Interviewees were aware and frustrated with this technological exceptionalism. Interviewee 5 described a recent seminar they had attended:

I’ve been in the room where…immediately at the end of the presentation [about the app]…everyone is asking lots of really hard questions about privacy, lots of questions about surveillance, lots of questions about technology. And then 15 minutes later, someone’s giving a talk about manual contact tracing…and not one question about privacy. So, I think there’s a technology exceptionalism going on.

This interviewee’s frustration, and the frustration of other interviewees was based on the perception that ethical debate associated with the app emerged from an ‘unethical’ (interviewee 2) privacy ‘lobby’ (interviewees 4, 5), which had ‘steered’ (interviewee 2) the media towards a polarised reporting of the app, solely in terms of a centralised/decentralised debate of ‘privacy diminishing versus privacy enhancing’:

the conversation got hijacked early on about centralised and decentralised, because there’s a very active privacy lobby in the UK and that’s just one of the facts of life when you try to do anything with data or technology (interviewee 4).

Interviewee 6 was particularly frustrated that, in their perception, this lobby had led to an ‘insane’ coverage of privacy debates in the media and policy, even though they perceived that they ‘bore no relationship to the actuality of the situation’ and were ‘divorced from reality’.

In fact, interviewees explained, the ethical debate associated with the app seemed to be focused on those ‘making the loudest noise’, which did not necessarily reflect public attitudes towards the app—attitudes that interviewees perceived had been shown to be overarchingly supportive of the technology (Abeler et al., 2020), For these interviewees, this lobby ‘wasn’t willing to let the public make its own decision’ (interviewee 5). In fact, professional interviewee 4 noted that they had spoken to an NHSX representative who had visited the Isle of wight on several occasions, and who had perceived residents to have little awareness or understanding of the decentralised/centralised debates*.* Our interviews with members of the Isle of Wight public suggested similar, with discussion about the decentralised/centralised distinction nearly invisible (unpublished).

Overall, then, all three sources of data- the news media, grey literature and professional interviewees -pointed to the perceived ethical distinction between centralised ‘privacy diminishing’ versus decentralised ‘privacy enhancing’ data collection approaches being the most prominent app-associated ethical issue discussed in the public and policy arena. Stakeholder interviewees reflected their perceptions that this focus on privacy issues had emerged from a ‘privacy lobby’.

### Other issues: transparency, accountability and the private sector’s role

Other ethical concerns relating to the app also featured in newspaper and policy discussions, though to a much lesser degree compared to the focus on centralised versus decentralised data collection approaches.

#### Openness and transparency

There was a strong sentiment across the news articles and grey literature, as well as from our stakeholder interviews, that there was a lack of transparency and communication from the government about the app’s development (‘there’s a question of transparency that has to do with what exactly it was implementing’ (interviewee 6)). The grey literature analysis highlighted how, while officials emphasised that they would maintain the highest level of transparency before and after the app was launched, commentators were concerned about the lack of information around data processing, creating suspicion about how the government was communicating to the public. This suspicion was also evident in the news articles. Stakeholder interviewees corroborated this lack of transparency, explaining that they, too, had noticed that the NHSX and the UK government minimally communicated with the public about the development of the app. As interviewee 8 remarked:

the Comms were awful, so the NHSX were completely silent about what they were doing and so everything was rumour. So there were leaks and rumour in the media. Instead of getting on the front foot and saying here’s what we’re building, here’s why we’re building it and here’s how it's going to work. You’ve 3 months of rumour and conjecture in the run up to the launch.

#### Involvement of Big Tech

Involvement, collaboration and/or competition with public and private corporations featured in the newspaper analysis, and to a lesser extent in the grey literature (Figs. 2, 3). This was particularly in relation to Apple and Google, who were sometimes discussed as allies (in terms of the decentralisted model of data collection), other times as obstacles (for instance, Apple not lowering its privacy safeguards with Bluetooth to accommodate centralised apps). Concerns were raised about many companies, such as Microsoft, Google, Palantir, Faculty, and Amazon forming partnerships with the NHS. In an open letter from a range of civil society organisations, privacy advocates and academic researchers published by Medium on 18th May 2020,^2^ it was concluded that since there was a lack of clarity on the nature of these partnerships and on how the NHS and the government would hold these partnerships accountable to data protection, the app was unlikely to meet appropriate data governance standards.

Furthermore, the relationship between the UK government and private corporations worried stakeholder interviewees. On one hand, they were anxious about the emerging power relationships between the two sectors. They spoke about the immense pressure coming from Apple/Google for the UK government to use their API, and in fact, how Apple had ‘refused’ (interviewee 5) to change a design feature that would have helped address an easily fixable issue associated with the app. Interviewee 5 remarked ‘[Apple/Google are] basically bullying governments into doing things that rightly or wrongly they didn’t want to do’.

On the other hand, they worried about the incentives the private sector brought to the app’s development. For example, the fact that decisions about the app software (e.g. the prioritisation of battery power versus public health utility) were being determined on incentives important to the private sector, rather than reflecting what was best for UK public health. As such, the values of these multi-national organisations were perceived to be written into the software. Interviewee 6 reflected, ‘whose ethics are we talking about here?’ while interviewee 8 commented:

what they really care about, it turns out, is battery life. So they didn't want to do anything that makes their battery last less long.…they have a different set of incentives to a public health authority..[and]..that's not necessarily the right balance in the UK or anywhere else for that matter but that's what they enforced. It’s enforcing values through software which is increasingly worrying.

For these interviewees, the UK government’s relationships with the private sector also raised broader concerns about the digital economy, and the fact that these issues, and the issues related to the huge amount of power the private sector was perceived to have, had not been addressed sufficiently in the public forum prior to COVID-19: ‘Google and Apple…already de facto had a huge amount of power…governments have allowed that to happen’ (interviewee 7).

### A missing debate on ‘societal impact ethics’

Ethical issues associated with the app’s use in society featured much less in the news or grey literature, and in some cases were completely absent from the discussion. Interviewee 3 reflected on their perceptions of this missing debate, explaining that these issues were perhaps ‘too far forward to consider’ during the technology’s development. As interviewee 1 explained about the Ethics Advisory Board’s discussions on the topic, they were only ‘giving advice on the development of the process’. Only interviewee 7 reflected on a range of societal impact-type issues;

I suppose the fears around the deployment would just be…“here is an algorithm that's decided that you may be at risk of spreading, of having it and spreading it”. And people being faced with awful decisions about “do I keep delivering food to my sick neighbour or do I fully isolate”. And there’s no way for them to know how accurate it is and “am I on the borderline between the risk”.

The only discussion about societal impact that featured in any detail in our sample of grey literature, and to a lesser extent news media, was related to the potential of app use to promote inequality through a digital divide, with not everyone having access to a smartphone, and elderly people more likely being excluded from use. In the grey literature, problems related to the potential for discrimination were raised shortly after the government announced the development of the app, and persisted throughout the timeframe of analysis. Some of our interviewees explained that these arguments lacked merit because contact tracing does not benefit the health of either the index case or their contacts, but rather protects the population at large by interrupting chains of virus transmission: app users do not benefit from the app and those at higher risk of dying from COVID-19 are more likely to benefit regardless of whether they have the app installed. However, this discussion also extended to concerns about the potential for employers or service providers to remove free liberties by denying access to employment or services if their app showed they should be isolating. The early-identified sources include an open letter signed by over 50 signatories published by Medium on 23rd March 2020^3^ and the Ada Lovelace Institute report *Exit through the App Store?* from 20th April (AdaLovelace Institute, 2020b). Multiple commentators urged for the forging of legislation to resolve these issues. Two draft coronavirus contact tracing bills were identified. In line with these commentators, interviewees 3 and 7 believed policy discussions about the digital divide did not go far enough because they perceived that such inequities could emerge into instances of infringements of people’s rights and discrimination. Interviewee 5, on the other hand, emphasised that not developing the app, could in fact lead to more inequality and that this ethics narrative had not been given the attention it deserved; ‘it’s not entirely clear to me that the…equity issues are worse using the app than not using the app…[..]..All of the discussions I was involved in were about the ethics of doing something…very, very little discussion about the ethics of not doing this’.

Overall, while the aforementioned ethics issues were discussed in our sample of both the news articles and the grey literature, they were debated to a much lesser extent than debates pertaining to the ethical (privacy) distinction between centralised versus decentralised app data collection approaches.

## Discussion

The sociology *of* ethics literature argues that social forces work to shape ethical issues, leading to some ethical debates being privileged, whilst others are marginalised (Williams & Wainwright, 2013). Our findings show how issues specifically relating to the ethical distinction between centralised versus decentralised contact tracing app data collection approaches received prominent coverage in the UK national newspapers and grey literatures that we analysed. The specific focus was around the perceived ‘privacy diminishing’ versus ‘privacy enhancing’ aspects. The sociology *of* ethics literature requires us to understand how social processes work to define what counts as ethical. Our interviewees suggested that there were specific networks that were composed of privacy advocates or ‘lobbyists’ who aimed to drive political and media discussion along a specific agenda. Our findings do not allow us to know whether such a lobby exists—more empirical data would be needed to determine this. Though perhaps a better way to understand the exceptional reporting around privacy concerns is, rather than viewing the issue as being related to a singular privacy lobby, relating it to the fact that privacy issues are one of the defining policy issues of our time (Bennett, 2008; Urwitz & Jaffer, 2020), and in the digital era, privacy concerns are even more omnipresent given the potential for personal data abuse, and have featured heavily in the media. Here, concerns about privacy—from matters of shielding specific behaviors from external interference (surveillance; e.g. see Zuboff, 2019), to protecting individual decision-making ability and controlling personal information (Bennett, 2008)—have been intensified by recent ‘big data’ media scandals. These have included the 2018 Cambridge Analytica scandal, the 2014 UK government public relations failure of care.data—an initiative that aimed to improve the use of General Practitioner data for research, but received harsh public criticism (Carter et al., 2015; Sterckx et al., 2016)—and the Google DeepMind, London Royal Free Hospital scandal, which involved the transfer of identifiable patient records across the entire Trust, without explicit consent, for the purpose of developing a clinical alert app for kidney injury (Powles & Hodson, 2017). Such scandals have brought to the forefront new concerns over the opacity of many digital surveillance practices and technologies (Roberts, 2019), and have highlighted further the growing authority of Big Tech corporations and ‘Big Data’ processing firms to assist governments in the collection, production, and presentation of publicly produced data for security purposes—another concern which also featured prominently in both the news and grey literature.

It is likely that the aforementioned ‘big data’ scandals, and the public narrative of concern around data protection, surveillance and other privacy issues, may have helped construct the social legitimacy for a focus on privacy in this instance, rather than a ‘privacy lobby’ per se (though, as stated above, we cannot discount the presence of a ‘lobby’ without further empirical analysis). Drawing on the performative ethics literature, because privacy issues have escalated over the past few decades (Ruohonen, 2019), and sit high in the public consciousness, these narratives (privacy concerns) were more likely to resonate and be understood by members of the public, who were more likely to buy-in and re-circulate them. This is because—as this literature suggests—ethics emerges out of a process which is rooted in people buying-in, mimicking, and circulating a performance around particular topics (e.g., echoing a narrative (Santis & Zavattaro, 2019).

The importance of discussing issues related to privacy should not be understated. At the same time, the sociology of ethics literature has shown how the social construction of ethical issues has led to some ethics narratives being marginalised (Petersen, 2005). We also saw this in our findings, where the public ethics narrative became predominated with a polarisation of privacy concerns premised around the decentralised and centralised modes of data collection for the contact tracing app (Hasselbalch & Tranberg, 2020). This is unsurprising because the news media likes to polarise debates (Seale, 2003). However, by focusing the public ethics narrative in this way, the newspaper and policy discussions created a moral future which had a limited ‘moral imagination’ to think through the full range of ethical issues associated with the UK NHSX contact tracing app (Coeckelbergh, 2007). We note two points related to this that emerged from our findings.

First, both news articles and grey literature constructed privacy in a narrow sense, focused on individual privacy. Scholars have argued that it is vital to understand that privacy is more than just the protection of individual privacy rights and data governance, but also includes ‘social privacy’, and is related to broader issues of capitalism and power, social/collective value, accountability and ethics (Bennett & Raab, 2020; Prainsack, 2020; Ruohonen, 2019). The way in which the de-centralised data collection method was framed as ‘privacy enhancing’ was therefore problematic. This is because the ‘enhancing’ aspect of privacy only considered one form of privacy—individual privacy rights. If we consider privacy in the broader, social sense, then we can see that there are other (social privacy) factors at play that question the assumption of this model as ‘privacy enhancing’. These other factors include aspects of power that emerge when considering the involvement of Big Tech, and specifically, issues relating to technology conglomerate power, and their increasingly infiltration into public sector technologies and infrastructure ((Hasselbalch & Tranberg, 2020); also Sharon, 2020). It is therefore important to acknowledge that while individual privacy is an important value, there are other values that also needed to be considered, not least the question of who stands to benefit from the privacy protection that was promised in the ‘privacy enhancing’ approach to de-centralised contact tracing apps. As, such, the notion of privacy was simplified to a false trade-off between an individual rights approach of a privacy enhancing and privacy diminishing option (Hasselbalch & Tranberg, 2020; Samuel, Chubb, et al., 2021), with the vested interests of the Big Tech giants, Apple and Google, while discussed, being marginalised in the ethical debate.

Second, other values beyond privacy are also important. Taking a sociology *of* ethics approach can expose missing debates, and indeed this is the main value of this approach. Indeed, the polarisation of issues in the public ethics narrative sat alongside the under-representation or absence of other ethical issues (Sharon, 2020). For example, there was an absence of discussion about the ethical issues associated with the societal impact (research/technology *use*) of the app. This was perhaps unsurprising given that issues associated with societal impact ethics were not included in the remit of the NHSX app’s Ethics Advisory Board (Samuel & Lucivero, 2021). Furthermore, the focus on privacy and data protection issues at the expense of research *use* ethics has also been seen in Higher Education Institution research ethics approval procedures for digital research (Samuel, Chubb, et al., 2021; Samuel, Roberts, et al., 2021). As has been argued there, this is problematic as it leads to ambiguity around who is responsible for monitoring the societal impact of technologies, especially because those who are responsible for designing technologies are not always responsible for implementation (Kerr et al., 2020; Samuel, Chubb, et al., 2021; Samuel, Roberts, et al., 2021). This ambiguity requires further attention. As Hasselbalch contests; ‘without a proper social impact ethics of the technologies we adopt today going beyond mere data protection and technical privacy, the consequences are dire’ (Hasselbalch & Tranberg, 2020; Hazaparu, 2014). For example, our (unpublished) interviews with members of the Isle of Wight public during the app trial highlighted a range of concerns important to them, but that featured less in policy and media discussions. These included their understanding of the app’s capabilities, and questions regarding how to use the app and respond to app instructions. Questions regarding when, where and how this ‘societal impact ethics’ should be debated remain unanswered, though there have been calls for an enforcement of an ex-post evaluation of technological research and development, at least in the health arena (Dawson et al., 2019; Samuel, Chubb, et al., 2021; Samuel, Roberts, et al., 2021). Such an evaluation could have perhaps recognised these concerns and assured that appropriate support was provided for members of the public during the Isle of Wight trial. It could perhaps also have considered how the privacy discourse compared to data on resulting harm (Laurie et al., 2014).

Overall, our findings highlighted an exceptionalising and narrowing of the public ethics debate associated with the UK contact tracing app around issues pertaining to the privacy diminishing versus privacy enhancing narrative associated with centralised and decentralised app data collection approaches. This created a limited ‘moral imagination’ of the different values and ethical issues associated with the UK NHSX contact tracing app (Coeckelbergh, 2007). Most prominently, it masked broader concerns related to the power of BigTech, as well as other concerns related to the societal impact of the technology. The implications of this are clear, and are seen in the unfolding of events related to the UK contact tracing app. Specifically, while technical issues were said to be the reason why the original UK COVID contact tracing app was remodelled using an API provided by Google/Apple, the technical issues were quickly fixed in the original app, suggesting that political concerns compounded by public attention/criticism of the so-called ‘privacy diminishing’ centralised model—i.e. the public ethics narrative—played a role in halting the trial (Samuel and Simms, *forthcoming*). Moving forward, by understanding that the extent to which the focus on an ethical issue is less based on an overarching set of principles than the mass appeal of its narrative (Santis & Zavattaro, 2019), policymakers would do better to mitigate the spread of unhelpful ethics narratives by allaying and engaging with stakeholder and public concerns. The UK government poorly communicated about the contact tracing app (Samuel, Chubb, et al., 2021). With no counter-narratives being disseminated in the public domain, it became more likely for members of the public to be bought-in by the ‘privacy narrative’ constructed in the public domain, which most likely played a role in the move to change the model of the app following the trial.

The limitations of this paper relate to how we defined ‘ethics’ during our coding, and the implications this may have had for our analysis. In addition, the fact that news media is now a multifaceted infrastructure, engaged with through a range of diverse platforms (including social media), all of which have a role to play in shaping of a public ethics discourse, whereas our analysis—because of time and funding resources—was limited to a national newspaper analysis. Furthermore, given the extensive use of contact tracing apps globally, further research should use our findings for a cross-country comparative analysis exploring how the public ethics narrative in different countries relates to a jurisdiction’s digital contact tracing landscape, if at all. The public ethics narrative in some European countries has already been analysed (Amann et al., 2021). Finally, given that contact tracing apps are now in everyday use in some jurisdictions, including the UK, it would be interesting to explore the public ethics debate longitudinally, as well as reflect on this in light of users’ experiences.

## Appendix: Interview schedule

### For non-EAB members

#### General

1. Can you tell me your background and how this led to your involvement in the NHSX contact tracing app?
2. Can you talk me through what your exact involvement in the app has entailed on a day-to-day basis?

#### Personal/professional views about the app

1. What do you see as the role of this app in the response to the pandemic?

#### Developing and implementing the app

1. Can you describe what technical or other issues, if any, you’ve come across (or know others have come across) during the development and/or implementation of the app?
2. If you are aware of this, how have these issues been addressed?
3. In your opinion, how have these challenges compared to those in other research or work that you/others do?

#### Making ethics decisions about the app

1. What broad ethical and/or social concerns or worries do you have about the app?
2. What reflections do you have on how the ethical issues related to the app compare to those related to contact tracing (non app related) more broadly?
3. What types of discussions have you been involved in, if any, that have talked about the ethical issues associated with the app?
4. What resources are you aware of—for example, guidelines, individuals, committees or organisations—that were drawn upon to support ethical decision making with regard to the app?
5. In your professional opinion, in these discussions, what decisions were made about the ethical issues and who made them?
6. Are there any aspects about the app that you feel felt, or felt, uneasy about from an ethical point of view? Why, why not?
7. In your opinion, do you feel the ethical governance of the app was adequate? Why, why not?
8. How would you have improved the ethical governance?
9. Could you describe the governance of the app (who answered to who, who made the decisions, did this change over time) and your reflections on it?
10. What are your reflections on how the governance of the app compares to that of the test and trace initiative?
11. In your opinion, how do you think the whole issue of the app has been dealt with by the government? What could the gov have done better?
12. Moving forward what are the best ways of addressing the concerns we have discussed in this interview?

### For EAB members

#### General

1. Can you tell me your background and how this led to your involvement in the EAB for the NHSX contact tracing app?
2. Can you talk me through what your exact involvement in the EAB is, and what this has entailed on a day-to-day basis?

#### Regarding the EAB

3. Could you describe to me how and when the EAB was set up?
4. Could you describe the remit of the EAB, how many times do you meet and who decides what to discuss?
5. What types of discussions have the EAB had?
6. What discussions have the EAB had, if any, regarding an exit strategy for the app?
7. Regarding the discussions the EAB have, how do the concerns about the app fit into the general test/trace strategy? What is exceptional about the app in this process? Why does it add an extra level of ethical concern? Should it?
8. Where does the EAB get their information from about the app to support your discussions, and what information is this?
9. What resources are you aware of—for example, guidelines, individuals, committees or organisations—that were drawn upon to support ethical decision making?
10. At the end of the EAB meetings, who writes the report and what happens to it?
11. Does the EAB have decision-making power, and if so, what?
12. Beyond the EAB, in your professional opinion who has made the final decisions about the app?
13. Finishing up talking about the EAB, what do you feel has worked and what hasn’t worked in the EAB?

#### Personal/professional views about the app

1. What do you see as the role of this app in the response to the pandemic?
2. What concerns or worries do you have about the app?
3. In your opinion, do you feel the oversight mechanism for the development of the app was adequate? Why, why not?
4. How would you have improved the oversight mechanism?
5. Do you know anything about the oversight mechanism that was put in place for the implementation of the app (e.g. evaluation beyond the technical issues)? Could you describe these to me?
6. In your opinion, and only if you feel comfortable talking about it, how do you think the whole issue of the app has been dealt with by the government? What could the gov have done better?
7. Moving forward what are the best ways of addressing the concerns we have discussed in this interview?

## Footnotes

1 See https://www.pressgazette.co.uk/uk-newspaper-and-website-readership-2018-pamco/; https://en.wikipedia.org/wiki/List_of_newspapers_in_the_United_Kingdom_by_circulation; https://www.thestudentroom.co.uk/showthread.php?t=1562617; https://www.statista.com/statistics/246077/reach-of-selected-national-newspapers-in-the-uk/.

2 https://medium.com/@anoukruhaak/open-letter-b7cb79832064.

3 https://rachelcoldicutt.medium.com/open-letter-contract-tracking-and-nhsx-e503325b2703.
